# An Index for Wine Acetaldehyde Reactive Potential (ARP) and Some Derived Remarks about the Accumulation of Acetaldehyde during Wine Oxidation

**DOI:** 10.3390/foods11030476

**Published:** 2022-02-06

**Authors:** Almudena Marrufo-Curtido, Vicente Ferreira, Ana Escudero

**Affiliations:** Laboratory for Aroma Analysis and Enology, Instituto Agroalimentario de Aragón (IA2-Unizar-CITA), Department of Analytical Chemistry, Faculty of Sciences, Universidad de Zaragoza, 50009 Zaragoza, Spain; amarrufo@unizar.es (A.M.-C.); vferre@unizar.es (V.F.)

**Keywords:** acetaldehyde, Fenton reaction, polyphenols, wine oxydation

## Abstract

The amount of acetaldehyde accumulated during wine oxidation was very small, far less than expected. The existence of polyphenols specifically reactive to acetaldehyde was postuled. In order to assess the acetaldehyde reactive potential (ARP) of wines, different reactive conditions have been studied: acetaldehyde concentration, temperature and pH. The evaluation/validation of developed ARP assay was made with 12 wines. Results have shown that high temperatures cannot be used to estimate wine ARP. In fact, at 70 °C acetaldehyde reacts strictly proportionally to wine total polyphenols. A reproducible index by letting wine at pH 2 react with 35 mgL^−1^ of acetaldehyde for 7 days was obtained and applied to 12 wines. Rosés did not consume any, whites consumed 8% and reds between 18 and 38% of their total acetaldehyde content. After pH correction, whites ARP can be similar to low ARP reds. Basic kinetic considerations derived from the measurement of ARP were applied to interpret observed acetaldehyde accumulation and consumption during the forced oxidation of the 12 wines. It is concluded that wine ARPs cannot explain the huge fraction of acetaldehyde presumably consumed by wine and the fraction of H_2_O_2_ produced during oxidation and not consumed by SO_2_ has to oxidize majorly wine components other than ethanol.

## 1. Introduction

The reactivity of acetaldehyde with polyphenols to form different structures is well-known from 1976 [1,2]. It was proposed through the carbocation of acetaldehyde and subsequently confirmed by Mass Spectrometry analysis with Inductively Coupled Plasma (ICP-MS) [3]. The formation of adducts mediated by ethyl bridge has been the object of study in many works; observing that it is more favored as the wine is more acidic [4] or the acetaldehyde concentration is higher [5,6]. Pyroanthocyanins are formed by a cycloaddition of acetaldehyde in enol form to anthocyanins such as malvidin-3-glucoside [7]. It has been evaluated how the formation of these polymers with acetaldehyde affects the quality of the wines in terms of color and astringency, mainly [8,9,10,11] in oxygenation in the course of wine-making as micro-oxygenation with the deliberate formation of acetaldehyde.

In recent papers in which a set of red wines [12] and synthetic wines containing polyphenols from grapes [13] were subject to a forced oxidation procedure [14], the amount of acetaldehyde accumulated was very small, far less than expected. Expectations were based on the common belief that, once SO_2_ is depleted, most of the H_2_O_2_ formed in the second reduction of O_2_, suffers a catalytic decomposition to form the hydroxyl radical known as Fenton reaction. Since this radical is so reactive, it was also assumed that the oxidation of ethanol was the major outcome. It is true that there are some reports demonstrating that wine cinnamic acids [15] and wine mercaptans [16] can quench the 1-hydroxyethyl radical. There are also some evidences showing that SO_2_ is not as reactive as expected. Amino acids can be degraded by oxidation in those conditions in which SO_2_ is theoretically available but the wine polyphenolic profile does not favor SO_2_ consumption [17]. So that it is apparent that wine contains some other antioxidants which can interfere not only with H_2_O_2_ but which may also anticipate to SO_2_. 

However, as the main wine oxidation mechanism proposed by Wildenradt and Singleton [18] has not been questioned in recent reviews [19,20,21,22] or text books [23] it is assumed that these reactions are not predominant.

Attending to these premises, it was assumed that the little accumulation of acetaldehyde was the consequence of the reaction between wine polyphenols and this aldehyde. Accordingly, the amount accumulated was normalized by the expected amount as an estimation of the fraction of unreacted acetaldehyde [12]. PLS models relating the size of those fractions to the wine chemical composition indicated that acetaldehyde accumulated in the presence of combined SO_2_ and in the absence of anthocyanins and some other polyphenols. The negative role for anthocyanins supported the relevance of the wine ARP (therein referred as aldehyde reactive polyphenols) in the limitation of levels of aldehydes in wine. Moreover, O_2_ consumption rates have been found to be negatively correlated to the wine content in acetaldehyde [17]. Such negative correlation was tentatively interpreted as the likely consequence of a higher ability some polyphenols specifically reactive to acetaldehyde to consume O_2_. 

All this evidence leads us to the possibility that the wine ARP plays an outstanding role in wine properties, determining the rate at which O_2_ is consumed and the ability of wine to accumulate aldehydes. Both are essential for the sensory quality [24] and longevity of the wines. Therefore, the goal of the present paper was to develop an analytical index to assess the ARP of wines and to check whether such potential can really justify the small amounts of acetaldehyde accumulated during wine oxidation.

## 2. Materials and Methods

### 2.1. Solvents and Chemicals

Sodium metabisulfite 99% (Na_2_S_2_O_5_), Fe (II) chloride (99%), tartaric acid (99%), sodium hydroxide (98%), sulphuric acid (96%), ortho phosphoric acid (85%), hydrogen peroxide 3% stabilized w/v VINIKIT, indicator 4,4, mixed (methyl red-methylene blue) VINIKIT, sodium hydroxide 0.01 molL^−1^ VINIKIT were from Panreac (Barcelona, Spain). Ethanol for gas chromatography analyses was purchased from Merk (Darmstadt, Germany). Acetonitrile of HPLC quality was obtained from Fluka Analytical (Buchs, Switzerland). Formic acid high purity grade was purchased from VWR Prolabo (Fontenay sous Bois, France). Acetaldehyde (≥99.5%) was supplied by Sigma-Aldrich (Madrid, Spain). 2,4-dinitrofenilhidrazina (DNPH) was supplied by Molekula (UK). Water was purified in a Milli-Q system from Millipore (Bedford, Germany).

### 2.2. Development of the ARP Assay

Wines used in these trials were all commercial and Spanish: 12 red wines (from different 2010–2018 vintages and from four different grape varieties Garnacha, Tempranillo, Merlot and Cabernet-Sauvignon) and two white wines (Viura 2016). Table 1 shows the samples corresponding to each test with some of its characteristics.

The analysis procedure was based on spiking commercial wines with different levels acetaldehyde inside of an oxygen-free chamber and analyzing it after a time. For this, wines were opened in an oxygen-free chamber from Jacomex (Dagneux, France), where they were fortified with acetaldehyde. For each test 3 independent vials without headspace were prepared. Once closed, the vials were removed from the oxygen-free chamber and sealed with silicone. The vials were incubated at 25, 45 and 70 °C (room temperature and 2 high temperatures to accelerate the reaction). Total acetaldehyde (from each replica) was analyzed by HPLC-UV. In all samples the concentration of total acetaldehyde was determined just after the initial addition of acetaldehyde (zero point) and after the corresponding sampling time.

#### 2.2.1. Assays at High Temperature

Wines have been spiked with 300 mgL^−1^ of acetaldehyde. Sampling times were the following: 1, 4, 5 and 7 days for the test at 70 °C.

#### 2.2.2. Assays at Intermediate and Room Temperatures

Wines have been spiked with 300 mgL^−1^ of acetaldehyde. Sampling times were the following: 1, 2, 4 and 7 days for the test at 25 and 45 °C.

#### 2.2.3. Assays at Lower pH

Two pH levels were tested: 1 and 2. The first thing was to bring all the wines to these pH values. Immediately afterwards, they were taken to the oxygen-free chamber and 3 independent vials (for each pH and wine) were fortified with 35 mgL^−1^ of acetaldehyde. Prepared samples were incubated at 25 degrees and the total acetaldehyde was analyzed by HPLC at time 0, 1, 2, 3, 4 and 7 days. The purpose was to find the sampling time with the maximum differentiation between samples.

The test was repeated at both pHs to evaluate the reproducibility of the procedure, this time sampling only at times that previously showed the highest differentiation between the samples (3 days for pH 1 and 7 days for pH 2).

### 2.3. Evaluation/Validation of the ARP Assay

In this assay, a set of 12 commercial wines was oxidized whose ARP capacity was previously evaluated by means of the previous index. The amount of acetaldehyde accumulated by each wine was measured too in order to check its relationship with the index.

Wines used in this experiment were all commercial and their characteristics appear in Table 1 and Table 2. pH value was determined by potentiometric method [25], total SO_2_ by the Rankine method following the recommendations of the OIV [26] and total polyphenol index (TPI) by spectrophotometric measurement at 280 nm as described by Ribéreau-Gayon et al. [27]. Iron was also determined by ICP-MS following the procedure described by Grindlay et al. [28]. ARPs index was determined in duplicate for all wines (at pH 2, fortified with 35 mgL^−1^ of acetaldehyde in strict anoxia and analyze the consumption of acetaldehyde after 7 days of incubation at 25 °C). Afterwards, each wine was introduced into the oxygen-free chamber where its iron levels were corrected, adding the necessary amount to bring them in all cases to the maximum level found in the set of samples. Wines were removed one by one and filtered through amicrobic filters of 73 mm diameter and 0.22 µm pore size (MERCK, REF: SCGP U02 RE, Damrstadt, Germany)). Once filtered, wines were saturated with air and placed in screw cap vials of 60 mL with the headspace adjusted to the volume strictly necessary to contain the level of O_2_ required to oxidize all the SO_2_ contained in that wine plus 35 mgL^−1^, as described in Marrufo-Curtido et al. [14]. For each wine, two independent vials were prepared with SPT3 sensors (Nomacorc) and incubated at 25 °C in a water bath with orbital shaking at 90 rpm. Dissolved oxygen levels were measured twice a day for the first week and then once a day, until the end of oxidation (at which point each tube had consumed 95% of available oxygen or after a maximum of 54 days of oxidation).

Once the oxidation was finished, for each vial the total acetaldehyde was analyzed by HPLC [29] and total SO_2_ by Rankine.

### 2.4. Determination of Total Acetaldehyde by HPLC

The determination of total acetaldehyde by HPLC-UV with previous derivatization with DNPH (2,4-dinitrophenylhydrazine) was carried out following the method described by Han et al. [29]. In this method, the sample is acidified with 25% sulfuric acid in order to favor the molecular form of the sulfur. In this form (molecular SO_2_) the sulfur does not form adducts with carbonyl compounds. Additionally, metabisulfite is added to the vial to prevent aldehyde formation during the derivatization process at 65 °C. Acetaldehyde at low pH is easily protonated and nucleophilic attack of the DNPH in the free and complexed forms of acetaldehyde, therefore quantifying the sum of both and obtaining the total acetaldehyde as a result. The calibration is carried out by injecting into each of the batches an unfortified synthetic wine and a fortified one with a known concentration of acetaldehyde.

### 2.5. Data Treatment for the Estimation of Wine ARP Index 

It is considered that the rate of consumption of acetaldehyde for the wine *i*, is governed by the following expression:

(1) $${\left(\frac{dC_{acet}}{dt}\right)}_{i}=-k_{ci}*{\left|ARPs\right|}_{i}*{\left|{\mathrm{CH}3\mathrm{CHOH}}^{+}\right|}_{i}$$

where $k_{ci}$ is a general kinetic constant integrating the different reactions of acetaldehyde with wine phenolics in wine *i*, ${\left|ARPs\right|}_{i}$ is the concentration of polyphenols reactive with acetaldehyde in that wine and ${\left|{\mathrm{CH}3\mathrm{CHOH}}^{+}\right|}_{i}$ is the concentration of the reactive cation derived from acetaldehyde. The expression can be related to wine pH and wine available acetaldehyde as follows:

(2) $${\left(\frac{dC_{acet}}{dt}\right)}_{i,\;pH}=-k_{ci}*{\left|ARPs\right|}_{i,\;pH}*\frac{\left|H^{+}\right|}{K_{a}}*{\left|\mathrm{CH}3\mathrm{CHO}\right|}_{i,\;pH}$$

where it is acknowledged that the availability of acetaldehyde is pH-dependent, that the reactivity of polyphenols also varies with pH, and that the proportion of reactive cation is a function of the pH, where Ka refers to the acid dissociation constant of the reactive cation (CH3CHOH+). To the best of our knowledge, such constant is unknown but it will be much higher than 1.

At pH 2 it will be considered that all acetaldehyde is available, so that,

$${\left|\mathrm{CH}3\mathrm{CHO}\right|}_{i,\;2}≅{\left(C_{\mathrm{CH}3\mathrm{CHOH}}\right)}_{i}$$

For one specific wine at pH 2, the three first factors in the right part of Equation (2) are constant. Therefore, by integration the following expression is derived:

(3) $${\left(\frac{C_{\mathrm{CH}3\mathrm{CHO}}^{t}}{C_{\mathrm{CH}3\mathrm{CHO}}^{o}}\right)}_{i}=e^{-k_{i\mathrm{pH}2}^{\prime }*t}$$

where the quotient is the one expressed, in percentage form, in Table 2, and $k_{i\mathrm{pH}2}^{\prime }$ is a pseudo constant for wine i which includes the kinetic constant, ${\left|ARPs\right|}_{i,\;2}$ and the quotient 10-2/Ka), and time is 7 days, the incubation time. This expression makes it possible to obtain an estimation of the pseudo first order kinetic constant of each wine at pH 2:

(4) $$k_{i\mathrm{pH}2}^{\prime }=\frac{1}{7}*ln\frac{C_{\mathrm{CH}3\mathrm{CHO}}^{o}}{C_{\mathrm{CH}3\mathrm{CHO}}^{t=7}}$$

Now, it will be assumed, just as a rough approximation, that the major effect of wine pH is on the concentration of the CH3CHOH+ cation (×10^2-pH^) so that at wine pH the kinetic constant of the pseudo first order process would become:

(5) $$k_{i\mathrm{pH}}^{\prime }=k_{i\mathrm{pH}2}^{\prime }*10^{\left(2-\mathrm{pH}\right)}$$

### 2.6. Statistical Analysis

Correlation studies and One-factor analysis of variance (ANOVA) were carried out with Excel 2016 (Microsoft, Washington, DC, USA). For significant effects, a Fischer post-hoc pairwise comparison (95%) test was performed.

## 3. Results and Discussion

### 3.1. Development of the ARP Assay

#### 3.1.1. Assays at High Temperature

The first obvious option to make an index for assessing the ability of a given wine to consume acetaldehyde is to rise temperature and acetaldehyde concentration. After some preliminary trials it became evident that, in order to get measurable consumptions in a reasonable time (few days), temperatures as high as 70 °C and levels of acetaldehyde as high as 300 mgL^−1^ had to be used. An additional advantage of large additions is that consumption becomes nearly independent of the wine SO_2_ level, which simplifies the procedure and the calculations. 

**Figure 1:**
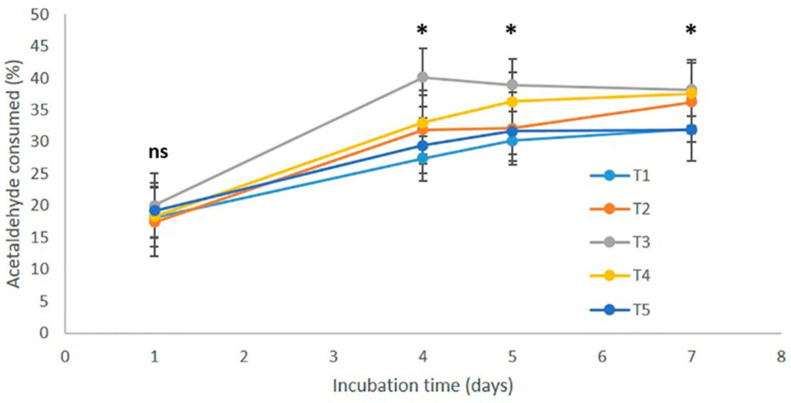
Evolution with time of the consumption of acetaldehyde (in %) by five different red wines (T1, T2, T3, T4 and T5) spiked with 300 mgL^−1^ of acetaldehyde. Wines were incubated in complete anoxia at 70 °C. Each experimental result is the average of 3 independent replicates. ns: not significant. *: significant differences (*p* < 0.05) each day.

Figure 1 summarizes the results obtained when 5 different red wines were spiked with 300 mgL^−1^ of acetaldehyde and incubated in complete anoxia at 70 °C increased times. The five wines used in this experiment were five reds from different ages and origins (Table 1), which supposedly, should have quite different ARPs. Results obtained showed, however, that all of them consumed acetaldehyde at quite similar rates. After one day of incubation at 70 °C, the % of acetaldehyde consumed by the five ranged from 17.4% to 20.0% with 18.6% as average. After 4 days, differences became maxima with consumptions between 27.4% and 40.1%. Afterwards, the samples seem to reach a plateau so that differences between samples begin to shrink. After 7 days of incubation, all the wines had consumed between 31.9% and 38.2% of the acetaldehyde added. The analysis of results revealed that the relative amounts of acetaldehyde consumed by each wine after 4 days of incubation were strictly proportional to the wine TPI (R = 0.9909, significant at *p* > 0.001). This result was certainly not expected, since in the previous experiment there was no relationship between wine TPI and its ability to accumulate acetaldehyde and Strecker aldehydes [12]. The conclusion, therefore, is that at high temperatures acetaldehyde reacts with all wine polyphenols in a non-discriminant way. For this reason, in the next experiments, lower temperatures were considered.

#### 3.1.2. Assays at Intermediate and Room Temperatures

**Figure 2:**
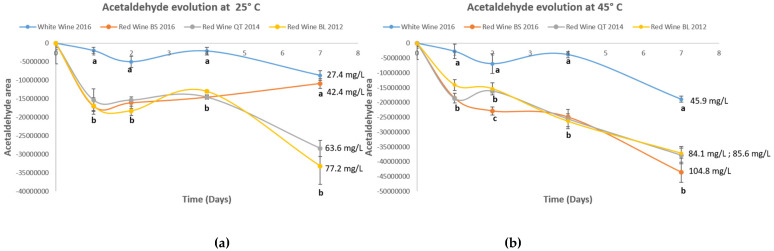
Evolution with time of the levels of acetaldehyde of four different wines spiked with 300 mgL^−1^ of this compound. Wines were incubated in complete anoxia at 25 °C (**a**) and 45 °C (**b**). Each experimental result is the average of 3 independent replicates. Number values indicate the consumed acetaldehyde (mgL^−1^). Different letters indicate significant differences (*p* < 0.05) each day.

Similar incubation experiments were carried out at 45 and 25 °C with a group of 4 quite different wines (3 reds with different ages and one white, Table 1). The wines were spiked with 300 mgL^−1^ of acetaldehyde and left to incubate up to 7 days in complete anoxia. The results are summarized in Figure 2. In general, it can be appreciated in both plots that the consumption of acetaldehyde is a little bit erratic at both temperatures. In both cases there is an apparent fast consumption during the first day, particularly relevant for red wines, followed by a plateau in which the consumption decreases or even stops, and a final period in which the consumption reactivates. Differences between both temperatures are mainly caused by the particular behavior followed by the red wine sample BS2016. This sample consumes acetaldehyde only the first day at 25 °C, so that ends the incubation period with a consumption close to that observed for the white wine. At 45 °C, however, it follows a consumption pattern similar to the two other red wines ending with the maximum amount of acetaldehyde consumed (Figure 2). Although the amount of acetaldehyde consumed at 45 °C was no longer correlated with wine IPT, the lack of correlation with the observations at 25 °C and the high homogeneity in acetaldehyde consumptions observed between wines (Figure 2), made us conclude that increasing temperature and acetaldehyde levels were not the best choice for measuring wine ARP. Although we did not observe any precipitation in the previous experiments, it has been described that large amounts of acetaldehyde can provoke the precipitation of polyphenolic material after longer incubation periods. This was observed, for instance, after incubating 80 days at 42 °C an equimolecular mixture of acetaldehyde and malvidin-3-*O*-glucoside [5]. Therefore, it can be thought that the high levels of acetaldehyde required for a reliable measurement of wine ARPs, destabilize wine polyphenolic material impeding a realistic measurement.

#### 3.1.3. Assays at Lower pHs

**Figure 3:**
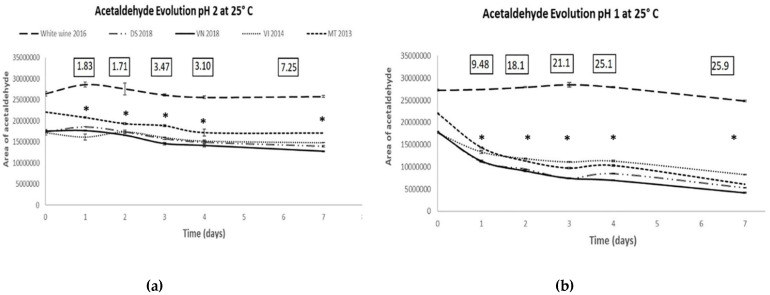
Evolution with time of the levels of acetaldehyde of five pH-adjusted different wines spiked with 35 mgL^−1^ of this compound. Wines were incubated in complete anoxia at 25 °C. Each experimental result is the average of 3 independent replicates. (**a**) Evolution in pH2 adjusted wines. (**b**) Evolution in pH1 adjusted wines. Boxed data, “discriminant index”, defined as the ratio “standard deviation of the means of different wines”/“average standard deviation within replicates”. *: significant differences between the four reds (*p* < 0.05) each day.

Since the main mechanism of reaction between acetaldehyde and wine polyphenols involves the protonated form of acetaldehyde [1,3], lowering the pH is an alternative way to accelerate the reaction. This strategy has been previously used by different authors to study different aspects of the reaction in wine models [6,30]. One additional advantage linked to the work at acid pHs is that the equilibrium of the reaction between acetaldehyde and SO_2_ is displaced towards the free forms. This makes it possible to study the kinetics in real wines avoiding the perturbation caused by the presence of SO_2_. To avoid the aforementioned possible problems introduced by large amounts of acetaldehyde, in this study wines were spiked with just 35 mgL^−1^ of acetaldehyde and left to react in complete anoxia at 25 °C for one week. The evolutions with time of the reaction for five different wines (four reds with different ages and one young white, Table 1) with pHs adjusted to 1.0 and 2.0 are given in Figure 3. As can be seen, the reaction is much quicker at pH 1. After 1 week, the four red wines had consumed more than 50% of the acetaldehyde. By comparison, at pH 2 the consumption after one week was above 25% in VN2018.

The potential quality of the indexes was measured by the “discriminant index”, defined as the ratio “standard deviation of the means of different wines”/“average standard deviation within replicates”. These ratios are given in the boxed data shown in the upper part of each plot (Figure 3). As can be seen, the highest ratios were obtained at pH 1, and at both pHs the indexes increase with time. Attending to the results given in the plots, 3 days at pH 1.0 or 7 days at pH 2.0 were retained for the study of reproducibility.

Reproducibility was checked by running two independent batches of triplicate samples for the five studied wines in different days. Results are summarized in Table 3. As can be seen, at pH 1 the assay was very repetitive within the same batch, but poorly reproducible between different batches, so that overall uncertainty was in the 12–25% range. At pH 2, repeatability was slightly worse, particularly for one of the batches of the oldest wine, but reproducibility between different batches was much better. It can be also observed that reproducibility for red young wines was outstanding (<2%), while for red aged wines it was just acceptable (<15%). It should be noted that in no case we observed turbidity or any precipitate, but the batch to batch imprecision suggests that the reaction could involve the formation and/or degradation of wine colloidal structures. In any case, the reaction at pH 2 with 7 days of incubation was chosen as the most adequate procedure for assessing the wine ARP.

### 3.2. Evaluation/Validation of the ARP Assay

Since the most evident expected effect of the wine ARP is to limit the accumulation of acetaldehyde during wine oxidation, the evaluation of the developed assay will check whether wine ARP measured with the index, can be effectively used to predict or explain the amount of acetaldehyde accumulated by the wines during oxidation. Attending expectations, wines with higher ARP should accumulate very little amounts of acetaldehyde, while those with low ARP, will accumulate acetaldehyde easily. For checking this, 12 different wines (2 whites, 2 rosés and 8 reds of different aging and phenolic characteristics, Table 1) were used. The ARP of the wines was measured with the developed index, and the wines were further subjected to a forced oxidation procedure [14].

#### 3.2.1. ARP Index of the Wines

The general properties of the wines used in the study, together with the results of the ARP index are given in Table 2. This table reveals that the amounts of acetaldehyde consumed during the ARP assay, listed in the column headed by “AR pH 2”, basically separate the wines by types. The two rosés did not consume any acetaldehyde in the week in which wines were incubated with 35 additional mgL^−1^ of acetaldehyde at pH 2. In one of the cases (sample 10), the amounts measured after the incubation were significantly higher than those measured before the experiment, so that a negative value is obtained. The two white wines consumed 4.7 and 8.3 mgL^−1^, while the reds consumed between 9.8 and 17.7 mgL^−1^. 

The column headed “% AR” gives these consumed amounts as percentages of the total acetaldehyde measured in the wines immediately after the incubation and after the spiking with 35 mgL^−1^ of acetaldehyde. This was necessary, since at pH 2 all acetaldehyde contained in the wine, including that originally present, should be available for reaction [31]. These figures are negative for rosés, are around 8% for the two whites, and range from 18.3% (sample 8) to 37.8% (sample 7) in the case of reds. It should be noted that the range of variability of this ARP index between red wines is not very wide. Although it is above a factor 2, the RSD% of these % AR for the 8 wines is just 24%. Furthermore, if samples 7 and 8 are excluded, the range goes from 20.5 to 27.8% with a RSD% for these 6 wines of just 12%. The range of variability is, anyway, higher than that of IPT (RSD11%). 

The column “k’_pH2_” is the natural logarithm of the inverse of this quotient (acetaldehyde concentration after 7 days/initial acetaldehyde concentration + 35 mgL^−1^) corrected by 7, the incubation time (Equation (4)) and corresponds to the pseudo first order kinetic constant for the reaction at pH 2. Assuming first order kinetics for acetaldehyde, this constant gives the fraction of the total acetaldehyde present reacted each day. It is, therefore, a value slightly higher than the seventh part of (%) AR. The last column in the table gives the pH-corrected pseudo first order constant at wine pH. This is just a rough estimation intending to provide an idea of the order of magnitude of the reaction rate at wine pH and in particular, of the differences between wines. The values for these k’_pH_ values suggest that because of the more acidic pHs, the acetaldehyde reactive capacity of whites should be comparable to those of some reds. Leaving aside the two rosés, k’_pH_ ranges from 0.00059 to 0.00117 with sample 7 as an outlier, which takes the value 0.0021.

#### 3.2.2. Forced Oxidation Procedure

Wines in Table 2 were further exposed to 35 mgL^−1^ of O_2_ plus the additional amount that by stoichiometry is required to completely oxidize their SO_2_. Wines were incubated at 25 °C the time required to consume 95% of the O_2_ supplied or to 54 days. The results of the experiment are given in Table 4. As can be seen, all red wines were able to consume the O_2_ provided before the maximum incubation time, while only one rosé and no white wines completed the consumption. In the case of reds, it is most remarkable that although young wines tend to consume O_2_ faster, the fastest consumption was observed in an aged wine. It is also noteworthy that the time required to consume the O_2_ was negatively correlated to wine pH (R = −0.743, *p* < 0.001) and positively correlated to wine initial total SO_2_ content (R = 0.734, *p* < 0.001). The negative effect of pH on the time required for consuming the O_2_ was expected, since the redox reaction leading to the formation of quinones from *o*-diphenols is pH-dependent and its potential decreases at higher pHs (the *o*-diphenol becomes a stronger reducer at higher pH). The positive dependence with SO_2_ was not expected, since it has been described that free SO_2_ is essential for O_2_ consumption, well by reducing back the quinone to the diphenol-form, well by reacting with it as nucleophile [32]. However, part of the correlation maybe just an artefact caused by the fact that wine SO_2_ content and wine pH bear a weak but significant negative correlation (R = −0.615, *p* < 0.05). Additionally, it should be considered that wine total acetaldehyde content is strongly correlated to its total SO_2_ content (WAcetaldehyde = −4.4 + 0.39 WtSO_2_; R = 0.837, *p* < 0.001) and wine acetaldehyde is also correlated to the time required to consume O_2_ (R = 0.669, *p* < 0.05). This is consistent with the negative correlation between total acetaldehyde and O_2_ consumption rate previously observed [17]. Such observation seemed to support the existence of a category of wine acetaldehyde reactive polyphenolics which would be also avid O_2_ consumers.

#### 3.2.3. Observed and Expected Accumulation of Acetaldehyde

The accumulation of acetaldehyde was in all cases very small. In fact, two of the wines (one aged red and one rosé) accumulated negative amounts; i.e., consumed acetaldehyde, and four more wines (one young red, the two white and one rosé) accumulated amounts not significantly different to 0. The maxima amount accumulated was 7.4 mgL^−1^ and in contrast to previously reported results [12], aged reds did not accumulate higher levels than young reds.

Furthermore, there are no clear correlations between the kinetic constants obtained in the ARP assay and the amount of acetaldehyde accumulated in the validation, taken into account or not O_2_ not SO_2_. The same results are obtained even inserting pH corrections in the kinetic constants, as shown in material and methods section. The kinetic constants calculated at wine pH are neither correlated with the acetaldehyde initial amounts (R = −0.19, *p* = 0.55), nor with the acetaldehyde accumulation normalized (R = 0.16, *p* = 0.62) or not (R = 0.20, *p* = 0.52) to “O_2_ not SO_2_” consumed in the oxidation.

Excluding white wines, the little amounts accumulated (or consumed in one case) are significantly correlated to the O_2_ not SO_2_ consumed by the wine (R = 0.836, *p* < 0.01) and are, consequently, equally correlated to the expected amounts of formed acetaldehyde. These expected amounts of acetaldehyde formed during the oxidation of each wine can be derived from the stoichiometric ratio between the “O_2_ not SO_2_” consumed and ethanol oxidized. For this, we will assume that all H_2_O_2_ produced in the oxidation is first transformed in hydroxyl radical, and that then 85% of this radical oxidizes wine ethanol, the most abundant organic compound, while the other 15% goes to oxidize other organic material (tartaric acid, polyphenols, glycerol) [33].

(6) $${\mathrm{AF}}_{\mathrm{expec}}=\frac{O_{2}{\mathrm{notSO}}_{2}}{32}*44*0.85=1.17*O_{2}{\mathrm{notSO}}_{2}$$

where AFexpec stands for acetaldehyde formed (expected). Note that this relationship assumes that any other reaction diverting H_2_O_2_ from the Fenton reaction, well by a direct reaction of H_2_O_2_, well by quenching the 1-hydroxylethyl radical, precursor of hydroxyl radical, is going to have a negligible effect. This is not strictly true, since there are at least two relatively recent reports demonstrating that wine cinnamic acids [15] and wine mercaptans [34] can quench the 1-hydroxyethyl radical. However, as the main wine oxidation mechanism proposed by Wildenradt and Singleton [18] remains for the most unquestioned, we will assume it is majorly true and will check whether the assumption is consistent with the experimental results. 

The relationship between acetaldehyde accumulated and the expected amounts of acetaldehyde formed and reacted will be given by:

AA = AFexpec − ARexpec(7)

So that, ARexpec = AFexpec − AA. These estimated values are given in the last columns of Table 4. As can be seen in the very last column, under the assumptions taken, a major fraction of the acetaldehyde formed by oxidation of ethanol would have reacted with wine polyphenols. Reacted values go from a 64% in the white wine 11, to values above 100% for the wines in which there was no accumulation of acetaldehyde, with most values within the range 81–96%.

Taking into account the wine pH and much lower acetaldehyde amount generated in the process, it should be expected acetaldehyde reaction rates more than one order of magnitude lower than those found in the ARP assay. The forced oxidation took place for more than 6 weeks. Neverthless, the acetaldehyde amounts that could be consumed by the wines according to acetaldehyde comsumption capacity shown by the ARP assay were much lower than those shown in the column ARexpec of Table 4.

It has to be therefore concluded that the main assumption made regarding the formation of acetaldehyde (Equation (6)) does not hold in any case. These means that reactions competing by H_2_O_2_ or by the 1-hydroxyethyl radical have to take significantly place during wine oxidation and that it cannot be longer considered that the major destiny of the H_2_O_2_ formed during oxidation is either the oxidation of SO_2_ or of ethanol. Further studies are required to estimate these mass balances and to identify the fate of the H_2_O_2_ produced during wine oxidation.

## 4. Conclusions

Temperatures higher than room temperature cannot be used to measure the wine ARP. At 70 °C, acetaldehyde reacts to wine polyphenols indiscriminately, so that reacted amounts after 4 days of reaction are proportional to wine TPI.

In terms of reproducibility a satisfactory assessment of wine ARP is obtained by the incubation of wine with pH adjusted to 2 with 35 mgL^−1^ of added acetaldehyde in strict anoxia for 7 days. Applied to 12 different wines, ARP separate wines by type. The 2 rosés were unable to react at this pH; the two whites consumed 8% of available acetaldehyde, while reds consumed between 18 and 38%. Correcting by pH, reactivity of whites can be similar to that of low ARP reds. Two samples of white wine and 2 samples of rosé wine have been used in this study. More samples of these types of wine should be studied to draw more powerful conclusions.

Levels of acetaldehyde accumulated by wines during oxidation are too low to be compatible with the common believe that most H_2_O_2_ not used to oxidize SO_2_ oxidizes ethanol. Basic kinetic considerations make it possible to conclude that wine ARPs cannot explain the huge fraction of acetaldehyde presumably consumed by wine associated to such assumption. Results suggest that most H_2_O_2_ has to oxidize other wine components. This fact should be taken into account in the control of the O_2_ consumption rate and of the accumulation of aldehydes in wines. Such a control is essential, for example, in the micro-oxygenation of wines, a very widespread technique in wineries, or in their aging. Explaining the O_2_ consumption rate and the accumulation of acetaldehyde is important to manage the longevity of wines and obtain wines with higher aromatic quality. 

## Figures and Tables

**Table 1:** Wines used in the development, evaluation and validation of the ARP assays.

N°	Code	Region	Variety	Vintage	TPI ^1^
	**Assay at 70 °C**				
1	T1 ^RDW 2^	Cariñena	Garnacha	2016	56.3
2	T2 ^RDW^	Rioja	Tempranillo	2015	50.2
3	T3 ^RDW^	Ribera del Duero	Tempranillo	2014	68.3
4	T4 ^RDW^	Toro	Tempranillo	2013	60.2
5	T5 ^RDW^	Navarra	M/C/T ^5^	2010	54.9
	**Assay at 25 and 45 °C**				
1	White wine	Rioja	Viura	2016	10.9
2	BS ^RDW^	Campo de Borja	Garnacha	2016	46.9
3	QT ^RDW^	Ribera del Duero	Tempranillo	2014	68.3
4	BL ^RDW^	Rioja	Tempranillo	2012	58.2
	**Assay at pH 1–2**				
1	White wine	Rioja	Viura	2016	5.2
2	DS ^RDW^	Mancha	Tempranillo	2018	41.1
3	VN ^RDW^	Ribera del Duero	Tempranillo	2018	42.9
4	VI ^RDW^	Cariñena	Garnacha	2014	48.9
5	MT ^RDW^	Toro	Tempranillo	2013	59.8
	**Evaluation/validation of the ARP assay**				
1	CV ^RDW^	Cariñena	G/T/Cr ^6^	2008	53.0
2	DM ^RDW^	Zamora	Tempranillo	2012	55.6
3	CL ^RDW^	Rioja	Tempranillo	2013	46.3
4	VF ^RDW^	Campo de Borja	Grenache	2015	54.7
5	MT ^RDW^	Toro	Tempranillo	2018	61.8
6	FP ^RDW^	Rioja	Tempranillo	2018	46.7
7	BG ^RDW^	Calatayud	Garnacha	2018	50.6
8	RB ^RDW^	Campo de Borja	Garnacha	2018	44.1
9	VMG ^RW 3^	Catalayud	Garnacha	2018	11.8
10	GF ^RW^	Navarra	G/T/M/C/Sy ^7^	2018	13.3
11	VT ^WHW 4^	Campo de Borja	Viura	2018	10.7
12	VM ^WHW^	Calatayud	Viura	2018	7.41

^1^ TPI (Total Pplyphenol Index); ^2^RDW (Red wine); ^3^ RW (Rosé Wine); ^4^ WHW (White wine); ^5^ M/C/T: Merlot-Cabernet Sauvignon-Tempranillo; ^6^ G/T/Cr: Grenache-Tempranillo-Cariñena; ^7^ G/T/M/C/Sy: Grenache-Tempranillo-Merlot-Cabernet Sauvignon-Syrah.

**Table 2:** Acetaldehyde Reactivity (AR). Basic properties of the wines used in the validation of the ARP index and measured ARP index of the wines.

Wines						ARP Index			
N°	Type ^1^	pH	Total SO_2_ (mgL^−1^)	Initial Acetald. ^2^ (mgL^−1^)	Initial Fe (mgL^−1^)	AR pH 2 ^3^ (mgL^−1^)	% AR ^4^	K’ pH 2 Kinetic Constant	K’ pH Kinetic Constant
1	AR	3.5	62.8	27.9	1.3	11.9	0.204	0.0326	0.00103
2	AR	3.8	20.0	8.76	1.2	11.2	0.268	0.0446	0.00071
3	AR	3.7	81.0	20.6	1.9	13.5	0.220	0.0355	0.00071
4	AR	3.5	124	53.8	1.2	17.7	0.220	0.0355	0.00112
5	YR	3.8	57.0	13.7	0.9	12.2	0.257	0.0425	0.00067
6	YR	3.6	52.0	20.1	1.8	14.5	0.278	0.0466	0.00117
7	YR	3.5	37.0	7.32	2.2	14.3	0.377	0.0677	0.00214
8	YR	3.4	96.0	20.7	1.3	9.8	0.183	0.0289	0.00115
9	YR1	3.3	86.0	26.0	0.4	−0.3	−0.005	−0.0007	−0.00004
10	YR1	3.2	78.0	30.5	2.1	−4.5	−0.080	−0.0110	−0.00069
11	YW	3.2	115	51.3	0.6	8.3	0.088	0.0132	0.00084
12	YW	3.3	97.0	22.2	0.2	4.7	0.079	0.0118	0.00059

^1^ AR (Aged Red), YR (Young Red), YR1 (Young Rose) and YW (Young white); ^2^ Content in total acetaldehyde of the original wine (previous to any spiking); ^3^ AR pH 2: Acetaldehyde reacted during the week in which the wine with pH at 2.0 spiked with 35 mgL^−1^ additional acetaldehyde was incubated in anoxia for 1 week; ^4^ % AR: quotient [“RA pH 2”/(initial acetaldehyde + 35)] × 100.

**Table 3:** Reproducibility of the ARP indexes a two different pHs. At pH 2, wines were incubated with 35 mgL^−1^ of acetaldehyde in anoxia for 7 days. At pH 1, the incubation time was 3 days. In each batch, 3 independent replicates of each sample were run. Different batches were prepared in different days. s: standard deviation.

	pH 2						pH 1					
	Batch 1		Batch 2				Batch 1		Batch 2			
Sample	Mean	s	Mean	s	Mean	%RSD	Mean	s	Mean	s	Mean	%RSD
White wine	2.60	1.38	0.0	1.54	1.30	99.8	−2.71	2.23	3.5	0.77	0.40	1100
DS ^RDW 1^	19.8	1.74	19.1	1.94	19.5	1.72	58.1	0.52	45.6	1.44	51.8	17.1
VN ^RDW^	27.2	1.07	27.8	0.28	27.5	1.14	58.3	0.97	68.9	0.35	63.6	11.7
VI ^RDW^	13.8	0.07	10.5	0.02	12.1	13.7	37.0	0.39	25.8	0.89	31.4	25.2
MT ^RDW^	22.5	1.52	17.9	17.8	20.2	11.5	54.9	0.545	44.7	0.02	49.8	14.5

^1^ RDW (Red wine).

**Table 4:** Results of the forced oxidation procedure applied to 12 different wines. Oxygen consumed, time required, SO_2_ consumed and remaining, O_2_ not SO_2_ (O_2_ not used to oxidize SO_2_), final acetaldehyde levels and acetaldehyde accumulated.

Nº	O_2_ Cons. (mgL^−1^)	Time (days)	SO_2_ Cons. (mgL^−1^)	SO_2_ Rem. (mgL^−1^)	O_2_ Not SO_2_ (mgL^−1^)	Final Acetaldehyde (mgL^−1^)	Accumulated Acetaldehyde AA (mgL^−1^)	Acetaldehyde Formed (Expected) ^1^ AFexp (mgL^−1^)	Acetaldehyde Reacted (Expected) ^2^ ARexp (mgL^−1^)	ARexp (%)
1	38.5	45.8	43.6	19.2	27.6	31	3.1	32.3	29.2	90%
2	36.7	23.8	8.8	11.2	34.5	14	5.24	40.3	35.1	87%
3	49.3	45.8	43.4	37.6	38.5	28	7.4	45.0	37.6	84%
4	49.8	51.8	108.8	15.2	22.6	44	−9.8	26.4	36.2	137%
5	43.1	30.8	37.6	19.4	33.7	19	5.3	39.4	34.1	87%
6	43.1	38.3	26.4	25.6	36.5	23	2.9	42.7	39.8	93%
7	40.0	38.8	15.4	21.6	36.1	12	4.68	42.2	37.5	89%
8	43.5	51.8	69.1	26.9	26.3	18	−2.7	30.7	33.4	109%
9	35.2	51.8	46.0	40.0	23.7	27	1	27.7	26.7	96%
10	33.1	53.8	46.5	31.5	21.5	26	−4.5	25.1	29.6	118%
11	27.2	53.8	54.2	60,8	13.7	57	5.7	16.0	10.3	64%
12	32.9	53.8	63.9	33.1	16.9	26	3.8	19.8	16.0	81%

^1^ AFexp: Acetaldehyde formed (expected); ^2^ ARexp: Acetaldehyde reacted (expected).

## Data Availability

Data is contained within the article.
